# Evaluating the Effectiveness of Screened Lactic Acid Bacteria in Improving Crop Residues Silage: Fermentation Parameter, Nitrogen Fraction, and Bacterial Community

**DOI:** 10.3389/fmicb.2022.680988

**Published:** 2022-05-24

**Authors:** Liwen He, Yimin Wang, Xiang Guo, Xiaoyang Chen, Qing Zhang

**Affiliations:** ^1^State Key Laboratory of Animal Nutrition, College of Animal Science and Technology, China Agricultural University, Beijing, China; ^2^College of Forestry and Landscape Architecture, Guangdong Province Research Center of Woody Forage Engineering Technology, Guangdong Key Laboratory for Innovative Development and Utilization of Forest Plant Germplasm, State Key Laboratory for Conservation and Utilization of Subtropical Agro-Bioresources, South China Agricultural University, Guangzhou, China

**Keywords:** bacterial community, lactic acid bacteria, peanut straw, silage quality, sweet potato vine

## Abstract

Ensiling characteristics of sweet potato vine (SPV) and peanut straw (PS), as well as the effects of lactic acid bacteria (LAB) strains, *Lactococcus Lactis* MK524164 (LL) and *Lactobacillus farciminis* MK524159 (LF), were investigated in this study. Fermentation parameters, nitrogen fractions, and bacterial community of SPV and PS were monitored at intervals during the ensiling process. The results showed that inoculating LAB increased lactate production (2.23 vs. 2.73%; 0.42 vs. 1.67% DM), accelerated pH decline (5.20 vs. 4.47; 6.30 vs. 5.35), and decreased butyrate (0.36% DM vs. not detected), ammonia-N (6.41 vs. 4.18% CP), or nonprotein-N (43.67 vs. 35.82% CP). Meanwhile, it altered the silage bacterial community, where the relative abundance of *Lactobacillus* was increased (6.67–32.03 vs. 45.27–68.43%; 0.53–10.45 vs. 38.37–68.62%) and that of undesirable bacteria such as *Clostridium, Enterobacter, Methylobacterium*, or *Sphingomonas* was much decreased. It is suggested that the screened LAB strains LL and LF can effectively improve the silage quality of SPV and PS silages.

## Introduction

According to the statistics, almost 92 million tons of sweet potatoes are produced annually, ranking second in the root and tuber crops grown in the world (FAOSTAT, 2020). Sweet potato vine (SPV) is the byproduct of sweet potato, accounting for ~64% of fresh biomass, with a forage yield as high as 14.6 tons/hectare (Aregheore, 2004; Claessens et al., 2009). As well, the peanut is an important cooking oil and protein-producing crop, and its global planting area is as large as 28.52 million hectares with an annual output of 45.95 million tons (FAOSTAT, 2020). It is estimated that peanut straw (PS) yield is also 60 to 65% of the biomass in peanut production (Zhao et al., 2012). China is the main producer of these two crops with a crop yield of 53 million and 17.73 million tons per year (FAOSTAT, 2020), necessarily with large biomass of their byproducts produced, which could be an important feed resource if well used. In practice, dietary inclusion of fresh SPV could promote follicular development of Chinese Meishan gilt and increase beneficial flora abundance (Xu et al., 2019; Zhang et al., 2019), and supplementing SPV to goats fed grass hay-based diet could improve growth performance and carcass traits, spare conventional concentrate, and improve production benefits (Tadesse et al., 2013). Likewise, PS contains about 60% total digestible nutrients and 14% crude protein along with medium fiber level (Sallam et al., 2019), and it could be used as a sole diet in goat feeding (Yusiati et al., 2016). From the above, exploiting these byproducts in animal feeding can not only relieve the shortage of feed resources but also promote animal growth.

However, a well storage method would be necessary to assure the nutrient preservation and year-round supply of such feed resources. Within the forage preservation methods, ensiling appears to be a more flexible and economic option compared with hay, especially in unfavorable weather seasons (Grant and Ferraretto, 2018). However, few research has focused on profiling their silage fermentation, and it might be a challenge to obtain quality silage given that these byproducts are commonly characterized as seasonal supply, high moisture, low nutrient content, and complicated bacterial community. Inoculating lactic acid bacteria (LAB) is a common way to promote the dominance establishment of LAB fermentation, thereby improving fermentation quality, nutrient preservation, and even aerobic stability. Shah et al. (2017, 2018, 2020, 2021) conducted a series of silage research with king grass, elephant grass, or hybrid pennisetum. The results showed that LAB inoculation could improve the fermentation quality, reduce undesirable microorganisms, and increase *in vitro* rumen gas production. Similarly, a previous study of our group showed that inoculating *Lactococcus Lactis* MK524164 (LL) and *Lactobacillus farciminis* MK524159 (LF) deriving from mature *Moringa oleifera* leaf silage could remarkably increase LAB abundance, lower silage pH value, and reduce dry matter loss in high-moisture (almost 80%) silage (Wang et al., 2019). Thus, it was hypothesized that inoculating LL and LF would improve the fermentation quality of SPV and PS silages. Besides, the effectiveness of LAB strains LL and LF in low-moisture silage had not been studied yet.

Accordingly, the objectives of this study were to (1) investigate the regular changes of SPV silage and PS silage, mainly paying attention to the dynamics of nitrogen distribution and microbial community; and (2) verify the effects of LAB strains LL and LF on improving silage quality of SPV and PS.

## Materials and Methods

### Experimental Design and Silage Preparation

Fresh SPV and PS were collected from the Qilin North test field of South China Agricultural University (Guangzhou, China) and then manually cut into ~2 cm length by a paper cutter. Subsequently, the prepared raw materials were separately subjected to ensiling treatments, that is, inoculating LAB strains *Lactococcus Lactis* MK524164 (LL) and *Lactobacillus farciminis* MK524159 (LF) or their mixture (1:1; MIX, only for SPV silage), along with the non-inoculated blank (CK). These two strains were previously screened and identified from *Moringa oleifera* leaf silage by the Gram stain, colony morphology, catalase activity test, and 16S rDNA sequencing. They can ferment cellobiose, maltose, sucrose, raffinose, inulin, and lactose, and were able to grow at pH 3.5 to 8.0 and at temperature 15°C to 40°C (Wang et al., 2019). To prepare inoculant powder of LL and LF, the stored strains (-80°C)were activated and incubated for 48 h in MRS broth at 37°C and then centrifuged to get the bacterial biomass, which was finally suspended with skimmed milk and lyophilized. The LAB number was enumerated on MRS agar.

For silage preparation, 200 g of forage was sprayed with given inoculant liquid (1 × 10^6^ colony forming units (CFU) per gram of fresh matter) or deionized water (10 ml), then sealed in a mini bag silo (20 by 30 cm) with a household food vacuum sealer (Lvye DZ280; Dongguan Yijian Packaging Machinery, Dongguan, China). Each treatment was individually prepared in 12 bags (3 replications × 4 sampling times), and totally 84 bags of silage were produced. During the ensiling fermentation (environmental temperature around 30°C), three bags of each treatment were randomly sampled on days 3, 7, 14, and 30.

### Ensiling Characteristic and Nitrogen Distribution Analysis

Each bag silo was sampled (10 g) in triplicate, of which one was used for microbial plate counting, one was prepared for pH and organic acids determination, and the last one was used for bacterial diversity analysis. Finally, the remaining silage was oven-dried at 65°C (48 h) for DM determination. In brief, the silage sample was soaked with sterilized saline and serially diluted, and then the supernatant was used to enumerate LAB, coliform bacteria, yeasts, and molds on Man Rogosa Sharpe (MRS) agar, Violet Red Bile agar, and Rose Bengal agar, respectively. Another sample was extracted with deionized water and filtered to determine pH, ammonia-N, and organic acids. The content of ammonia-N was determined by the colorimetric method of Broderick and Kang (1980). The concentrations of lactic acid, acetic acid, propionic acid, and butyric acid were measured using high-performance liquid chromatography (HPLC) equipped with Shodex RSpak KC-811S-DVB gel C column (8.0 mm × 30 cm; Shimadzu, Tokyo, Japan) and SPD-M10AVP detector, under the conditions of oven temperature 50°C, mobile phase 3 mmol/L HClO_4_, flow rate 1.0 ml/min, and injection volume 5 μl. The dried sample was ground to pass a 1-mm screen and then used to analyze crude protein (CP) and true protein (TP) using automatic Kjeldahl apparatus (Kjeltec 8400, FOSS), where nonprotein-N was calculated by the difference of CP and TP. The contents of neutral detergent fiber (NDF) and acid detergent fiber (ADF) were determined by an A220 Fiber Analyzer (ANKOM Technology Corp., Macedon, NY, USA), and water-soluble carbohydrates (WSC) content was analyzed with a commercial test kit using the anthrone-sulphuric acid method. All detailed procedures of these analyses referred to He et al. (2019).

### 16S rDNA Sequencing Analysis of Bacterial Community

The sample was submitted for 16S rDNA sequencing in Guangzhou Gene Denovo Co. Ltd. (Guangzhou, China), and the detailed procedures were the same as He et al. (2019). In brief, bacterial DNA was extracted with DNA Kit (Omega Biotek, Norcross, GA, USA). Then, PCR amplification was conducted with the primers (341F: CCTACGGGNGGCWGCAG; 806R: GGACTACHVGGGTATCTAAT) targeting at V3-V4 regions of 16S rDNA. After purification and quantification, amplicons were sequenced using Illumina Hiseq 2500. As for data analysis, any sequence that contained over 10% of unknown nucleotides (N) or <80% of bases with Q value >20 were removed using FASTP to obtain clean reads, which were then merged as raw tags using FLSAH (v 1.2.11) with the settings of minimum overlap 10 bp and mismatch error rates 2%. Raw tags were denoised using QIIME (V1.9.1) pipeline and chimera-filtered with the UCHIME algorithm. Operational taxonomic units (OTUs) were clustered using the UPARSE pipeline at the similarity level of 97%. Taxonomic classification at the phylum and genus levels was conducted using the RDP classifier (Version 2.2) based on the SILVA database. The alpha-diversity indices covering Sobs, Shannon, Simpson, Chao, Ace, and Goods_coverage were calculated in QIIME (V1.9.1). Principal coordinate analysis (PCoA) of unweighted UniFrac distances and relative abundance comparison was performed in software R. The datasets presented in this study can be found in online repositories. The names of the repositories and accession number(s) can be found below: [PRJNA812632; PRJNA813727] https://www.ncbi.nlm.nih.gov/bioproject/?term=PRJNA812632 or PRJNA813727.

### Statistical Analysis

The effects of ensiling time, LAB inoculant, and their interaction on ensiling characteristics and nitrogen fractions were analyzed using two-way ANOVA on SAS 9.3 with the statistical model:


$$\begin{array}{l} {\text{Y}}_{\text{ij}}=\text{μ}+{\text{D}}_{\text{i}}+{\text{T}}_{\text{j}}+{\left(\text{D}\times \text{T}\right)}_{\text{ij}}+{\text{e}}_{\text{ijk}} \end{array}$$

where Y_ijk_ was every observation; μ was the general mean; D_i_ denoted the effect of ensiling time day i; T_j_ represented the effect of LAB inoculant j; (D × T)_ij_ accounted for the interaction of ensiling time day i and LAB inoculant j; and e_ijk_ was random residual error. Duncan's test was used to do multiple comparisons and statistical significance was declared when *P* < 0.05. An online platform (http://www.omicshare.com/tools) was used to analyze the sequencing data of the bacterial community.

## Results

### Characteristics of Raw Sweet Potato Vine and Peanut Straw

The characteristics of raw SPV and PS are shown in Table 1. The DM contents of SPV and PS used in this study were separately 11.28 and 48.71, and their CP contents were 13.44 and 9.96% (with true protein proportion of 79.20 and 87.01% CP), along with moderate fiber content (42.19% NDF, 30.14% ADF and 39.44% NDF, 20.75% ADF). The WSC content was 3.92 or 10.35% for SPV and PS, and their plate counts of LAB, coliform bacteria, molds, and yeasts were 5.47, 5.84, 4.23, 4.33 and 3.60, 5.43, 4.31, 4.43 log_10_ CFU/g FM, respectively. Furthermore, 16S rDNA sequencing analysis of the bacterial community revealed that the Sobs, Shannon, Simpson, Chao, Ace, and Goods_coverage of the bacterial community in SPV and PS silages were 918, 3.46, 0.74, 1484, 1535, 0.995 and 1077, 1.57, 0.31, 1960, 2022, 0.994, respectively.

**Table 1:** Characteristics of raw sweet potato vine and peanut straw.

**Item**	**Sweet potato vine**	**Peanut straw**
Dry matter (%)	11.28 ± 0.20	48.71 ± 0.17
Crude protein (% DM)	13.44 ± 1.10	9.96 ± 0.62
True protein (%DM)	10.64 ± 0.24	8.67 ± 0.19
Neutral detergent fiber (% DM)	42.19 ± 2.30	39.44 ± 1.96
Acid detergent fiber (% DM)	30.14 ± 1.44	20.75 ± 1.32
Water soluble carbohydrates (% DM)	3.92 ± 0.23	10.35 ± 0.79
Lactic acid bacteria (log_10_ CFU/g FM)	5.47 ± 0.43	3.60 ± 0.43
Coliform bacteria (log_10_ CFU/g FM)	5.84 ± 0.18	5.43 ± 0.91
Molds (log_10_ CFU/g FM)	4.23 ± 0.43	4.31 ± 0.15
Yeasts (log_10_ CFU/g FM)	4.33 ± 0.50	4.43 ± 0.10
Sobs	918 ± 30	1,077 ± 25
Shannon	3.46 ± 0.24	1.57 ± 0.10
Simpson	0.74 ± 0.03	0.31 ± 0.03
Chao	1,484 ± 53	1,960 ± 158
Ace	1,535 ± 101	2,002 ± 125
Goods_coverage	0.995 ± 0.001	0.994 ± 0.001

### Ensiling Characteristics of Inoculated or Non-Inoculated SPV and PS Silages

The dynamic changes in pH value, individual organic acid content, and microbial population are presented in Table 2. The application of inoculant LL, LF, or MIX decreased (*P* < 0.05) the pH values of SPV and PS silages, especially at the early stage. Consistently, inoculating LAB increased (*P* < 0.01) initial LAB loading and promoted (*P* < 0.01) acid production (lactic acid), thus accelerating (*P* < 0.01) pH decline. In addition, the growth of coliform bacteria was inhibited, and yeasts and molds, as well as propionic acid and butyric acid, were not detected in the inoculated SPV and PS silages.

**Table 2:** The effect of LAB inoculation on the fermentation characteristics of SPV and PS silages.

**Item**	**Sweet potato vine silage**					***P*** **-value**				**Peanut straw silage**				***P*** **-value**			
	**Trt**	**Day 3**	**Day 7**	**Day 14**	**Day 30**	**SEM**	**D**	**T**	**DT**	**Day 3**	**Day 7**	**Day 14**	**Day 30**	**SEM**	**D**	**T**	**DT**
DM	CK	11.48^a^	11.33^a^	10.48^ab^	10.06^b^	0.25	**	NS	NS	47.81^b^	49.13^a^	49.24^a^	49.41^a^	1.41	*	NS	NS
	LL	11.10^ab^	11.34^a^	10.92^ab^	10.65^b^					48.47	48.32	49.33	49.18				
	LF	11.38^a^	11.49^a^	10.67^b^	10.42^b^					48.51^b^	50.34^a^	49.78^ab^	50.40^a^				
	MIX	11.14^ab^	11.52^a^	10.40^b^	10.64^b^												
pH	CK	5.20^A^^a^	4.82^A^^ab^	4.50^b^	4.58^A^^b^	0.07	**	**	**	6.30^A^^a^	6.24^A^^a^	5.40^A^^b^	5.53^A^^b^	0.02	**	**	**
	LL	4.59^B^^a^	4.38^B^^b^	4.39^b^	4.38^AB^^b^					5.40^B^^a^	4.49^B^^b^	4.39^B^^b^	4.26^B^^b^				
	LF	4.52^B^^a^	4.42^B^^ab^	4.38^b^	4.38^AB^^b^					5.35^B^^a^	4.57^B^^b^	4.36^B^^bc^	4.28^B^^c^				
	MIX	4.47^B^^a^	4.45^AB^^a^	4.37^ab^	4.30^B^^b^												
LAB	CK	7.93^B^^a^	8.00^a^	7.68^ab^	6.80^B^^b^	0.16	**	**	NS	6.67^B^^c^	7.84^B^^ab^	8.57^B^^a^	7.06^C^^b^	0.04	**	**	**
	LL	8.84^A^^a^	8.20^ab^	7.77^b^	7.37^AB^^b^					8.86^A^^b^	9.69^A^^a^	9.42^A^^a^	8.09^B^^c^				
	LF	8.67^AB^^a^	8.29^ab^	7.71^b^	7.19^AB^^b^					8.88^A^^b^	9.53^A^^a^	9.30^A^^a^	9.17^A^^ab^				
	MIX	8.98^A^^a^	8.32^ab^	7.70^b^	7.63^A^^b^												
Coli.	CK	6.25	2.47	<2.00	<2.00	–	–	–	–	4.83	4.03	2.49	3.58	–	–	–	–
	LL	4.25	<2.00	<2.00	<2.00					<2.00	<2.00	<2.00	<2.00				
	LF	3.23	<2.00	<2.00	<2.00					3.89	<2.00	<2.00	<2.00				
	MIX	3.47	<2.00	<2.00	<2.00												
LA	CK	1.93^B^^b^	2.32^B^^ab^	2.75^B^^a^	2.23^B^^b^	0.09	**	**	NS	0.04^C^^d^	0.13^C^^c^	0.55^C^^a^	0.42^B^^b^	0.24	**	**	**
	LL	2.47^A^^a^	2.83^A^^a^	2.77^B^^a^	2.55^AB^^ab^					0.45^B^^d^	1.39^A^^c^	1.54^A^^b^	1.67^A^^a^				
	LF	2.50^A^^b^	2.97^A^^a^	2.91^A^^a^	2.62^AB^^b^					0.66^A^^c^	1.21^B^^b^	1.35^B^^b^	1.58^A^^a^				
	MIX	2.54^A^^b^	2.71^AB^^b^	3.09^A^^a^	2.73^A^^b^												
AA	CK	0.51^b^	1.36^ab^	1.60^a^	1.82^a^	0.14	**	0.44	NS	ND	ND	0.03	0.03	–	–	–	–
	LL	0.88^b^	0.96^b^	1.26^ab^	1.51^a^					ND	0.15	0.24	0.29				
	LF	0.92^b^	0.99^b^	1.38^ab^	1.64^a^					ND	0.13	0.22	0.30				
	MIX	0.80^b^	1.17^ab^	1.49^a^	1.42^a^												
BA	CK	ND	0.13	0.13	0.36	–	–	–	–	ND	ND	ND	ND	–	–	–	–
	LL	ND	ND	ND	ND					ND	ND	ND	ND				
	LF	ND	ND	ND	ND					ND	ND	ND	ND				
	MIX	ND	ND	ND	ND												

*LAB (log_10_ CFU/g fresh matter), lactic acid bacteria; SPV, sweet potato vine; PS, peanut straw; CK, non-inoculated silage (Control); LL, silage inoculated with Lactococcus lactis (LL); LF, silage inoculated with Lactobacillus farciminis (LF); MIX, silage inoculated with the mixture (1:1) of LL and LF*.

*DM (%), dry matter; LA (% DM), lactic acid; AA (% DM), acetic acid; BA (% DM), butyric acid; Coli., coliform bacteria; SEM, standard error of means; D, the effect of ensiling time; T, the effect of LAB inoculation; DT, the interaction of ensiling time and LAB inoculation; ND, not detected; “–”, default. The population of yeast and mold was <2 log_10_ CFU/g fresh matter, and propionic acid was not detected in this study*.

A−C *Means in the same column followed by different uppercase letters differ (P < 0.05);*

a−c *Means in the same row followed by different lowercase letters differ (P < 0.05)*.

“^*^ and ^**^” denotes P < 0.05 and P < 0.01, respectively, and NS means P > 0.05.

The nitrogen fractions covering crude protein, true protein, non-protein-N, and ammonia-N of non-inoculated/inoculated SPV and PS silages are summarized in Table 3. In this study, the CP content of SPV or PS silage was not affected (*P* > 0.05) by LAB inoculation, but the CP content of SPV silage was decreased when compared to that of fresh SPV. The proportion of true protein decreased (*P* < 0.01) during the ensiling process, which was inhibited (*P* < 0.01) by inoculating LAB for PS silage but not for SPV silage. Meanwhile, ammonia-N proportion gradually increased (*P* < 0.01), and LAB inoculation decelerated (*P* < 0.01) its production in SPV silage, while PS silage contained a low ammonia-N proportion.

**Table 3:** The effect of LAB inoculation on the nitrogen distribution in SPV and PS silages.

**Item**	**Sweet potato vine silage**					***P*** **-value**				**Peanut straw silage**				***P*** **-value**			
	**Trt**	**Day 3**	**Day 7**	**Day 14**	**Day 30**	**SEM**	**D**	**T**	**DT**	**Day 3**	**Day 7**	**Day 14**	**Day 30**	**SEM**	**D**	**T**	**DT**
CP	CK	12.81^a^	12.43^a^	11.50^b^	11.12^b^	0.62	*	NS	NS	9.73	9.63	10.42	9.67	0.78	NS	NS	NS
	LL	12.14^a^	11.32^ab^	11.17^ab^	10.52^b^					10.11	10.56	10.33	9.64				
	LF	12.65^a^	11.65^ab^	11.40^b^	11.30^b^					9.87	10.12	10.34	10.21				
	MIX	12.40^a^	10.63^b^	10.24^b^	10.46^b^												
TPR	CK	66.38^A^^a^	60.28^AB^^b^	56.82^AB^^c^	55.33^AB^^c^	1.61	**	**	*	68.26^B^^a^	65.11^B^^a^	55.78^C^^b^	56.33^B^^b^	4.93	**	**	*
	LL	67.74^A^^a^	58.42^B^^b^	57.88^A^^b^	56.19^A^^c^					72.22^A^^a^	67.67^A^^b^	61.45^B^^c^	61.56^A^^c^				
	LF	68.05^A^^a^	62.02^A^^b^	57.26^A^^c^	53.98^B^^d^					71.78^A^^a^	65.81^AB^^b^	64.11^A^^b^	64.22^A^^b^				
	MIX	62.18^B^^a^	59.72^B^^ab^	55.01^B^^b^	49.90^C^^c^												
NPNR	CK	33.62^B^^c^	39.72^AB^^b^	43.18^AB^^a^	44.67^BC^^a^	1.61	**	**	*	31.67^A^^c^	34.93^A^^b^	44.53^A^^a^	43.67^A^^a^	4.93	**	**	*
	LL	32.26^B^^c^	41.58^A^^b^	42.12^B^^b^	43.81^C^^a^					27.78^B^^c^	32.33^B^^b^	38.55^B^^a^	38.40^B^^a^				
	LF	31.95^B^^d^	37.98^B^^c^	42.74^B^^b^	46.02^B^^a^					28.22^B^^b^	34.22^A^^a^	35.89^C^^a^	35.82^B^^a^				
	MIX	37.82^A^^d^	40.28^A^^c^	44.99^A^^b^	50.10^A^^a^												
NHR	CK	1.79^A^^c^	3.99^A^^b^	5.90^A^^a^	6.41^A^^a^	0.72	**	**	NS	0.24^d^	0.38^c^	0.48^b^	0.63^a^	0.09	**	NS	NS
	LL	1.38^AB^^b^	1.90^B^^b^	3.67^B^^a^	4.18^B^^a^					0.20^c^	0.35^b^	0.48^b^	0.72^a^				
	LF	0.99^B^^d^	2.31^B^^c^	4.20^B^^b^	5.43^AB^^a^					0.23^c^	0.39^b^	0.51^b^	0.74^a^				
	MIX	1.00^B^^b^	2.24^B^^b^	5.50^A^^a^	5.64^AB^^a^												

*LAB, lactic acid bacteria; SPV, sweet potato vine; CK, non-inoculated SPV silage (Control); PS, peanut straw; LL, silage inoculated with Lactococcus lactis (LL); LF, silage inoculated with Lactobacillus farciminis (LF); MIX, silage inoculated with the mixture (1:1) of LL and LF*.

*DM (%), dry matter; CP (% DM), crude protein; TPR (% CP), the proportion of true protein; NPNR (% CP), the proportion of nonprotein-N; NHR (% CP), the proportion of ammonia-N (protein equivalent); SEM, standard error of means; D, the effect of ensiling time; T, the effect of LAB inoculation; DT, the interaction of ensiling time and LAB inoculation*.

A−C *Means in the same column followed by different uppercase letters differ (P < 0.05)*;

a−d *Means in the same row followed by different lowercase letters differ (P < 0.05)*.

“^*^ and ^**^” denotes P < 0.05 and P < 0.01, respectively, and NS means P > 0.05.

### Bacterial Community Succession of Inoculated or Non-Inoculated SPV and PS Silage

The alpha-diversity indices covering Sobs, Shannon, Simpson, Chao, Ace, and Goods_coverage of the bacterial community in SPV and PS silages are summarized in Table 4. The Shannon, Chao, and Ace of the bacterial community in SPV silage varied (*P* < 0.05) during the ensiling process, while the Sobs, Shannon, Simpson, and Goods_coverage of that in PS silage were different (*P* < 0.05) at various time-points. As to the treatments, the bacterial community of inoculated SPV silage had higher (*P* < 0.01) Chao and Ace as well as lower (*P* < 0.01) Goods_coverage relative to the control, while that of inoculated PS silage was lower (*P* < 0.01) in Shannon, Simpson, and Goods_coverage. In addition, there existed an interaction effect (*P* < 0.01) of ensiling time and inoculation on these alpha-diversity indices of the bacterial community in PS silage.

**Table 4:** The effect of LAB inoculation on the alpha-diversity of bacterial community in SPV and PS silages.

**Item**	**Sweet potato vine silage**					***P*** **-value**				**Peanut straw silage**				***P*** **-value**			
	**Trt**	**Day 3**	**Day 7**	**Day 14**	**Day 30**	**SEM**	**D**	**T**	**DT**	**Day 3**	**Day 7**	**Day 14**	**Day 30**	**SEM**	**D**	**T**	**DT**
Sobs	CK	1,177	1,001	940	1,182	84	NS	NS	NS	1,232^B^^b^	1,499^ab^	1,691^A^^a^	1,441^A^^ab^	62	*	NS	**
	LL	1,202	1,170	1,224	1,444					1,537^A^	1,404	1,339^B^	1,233^B^				
	LF	1,172	1,176	1,021	1,183					1,399^AB^^b^	1,514^a^	1,369^B^^bc^	1,329^AB^^c^				
	MIX	1,302	1,087	1,223	1,220												
Shannon	CK	5.58	5.58^A^	5.14	5.68	0.33	**	NS	NS	2.57b	4.30^A^^a^	4.09^A^^a^	4.32^A^^a^	0.17	*	**	**
	LL	4.98	4.91^B^	4.96	6.01					2.92a	2.69^B^^ab^	2.48^B^^bc^	2.44^B^^c^				
	LF	4.87	4.79^B^	5.34	5.63					2.89	2.93^B^	2.42^B^	2.61^B^				
	MIX	5.02^b^	4.50^B^^b^	5.08^b^	5.90^a^												
Simpson	CK	0.94	0.95	0.92	0.93	0.02	NS	NS	NS	0.51^B^^b^	0.79^A^^a^	0.81^A^^a^	0.78^A^^a^	0.02	*	**	**
	LL	0.90	0.91	0.90	0.92					0.68A^a^	0.66^B^^a^	0.56^B^^c^	0.61^B^^b^				
	LF	0.89	0.91	0.94	0.93					0.68^A^^a^	0.61^B^^ab^	0.56^B^^b^	0.58^B^^b^				
	MIX	0.91	0.88	0.91	0.95												
Chao	CK	1,649^ab^	1,529^B^^ab^	1,432^B^^b^	1,758^a^	85	*	**	NS	1,889^B^^b^	2,317^a^	2,631^A^^a^	2,305^a^	112	NS	NS	**
	LL	1,846	1,698^A^	1,767^AB^	1,868					2,361^AB^	2,540	2,282^B^	2,347				
	LF	1,789	1,751^A^	1,563^AB^	1,700					2,594^A^^a^	2,400^ab^	2,320^B^^b^	2,197^b^				
	MIX	1,979^a^	1,627^AB^^b^	1,865^A^^ab^	1,768^ab^												
Ace	CK	1,689^B^	1,537^B^	1,442^B^	1,734	82	*	**	NS	1,923^B^^b^	2,328^a^	2,634^A^^a^	2,297^a^	99	NS	NS	**
	LL	1,785^AB^	1769^A^	1595A^B^	1705					2345^A^	2505	2305^B^	2450				
	LF	1,870^AB^	1,773^A^	1,817^A^	1,890					2,686^A^^a^	2,496^ab^	2,332^B^^bc^	2,218^c^				
	MIX	1,990^A^^a^	1,654^AB^^b^	1,858^A^^ab^	1,741^ab^												
Goods_ coverage	CK	0.995^A^	0.995^A^	0.995^A^	0.994	0.000	NS	**	NS	0.995^A^^a^	0.993^ab^	0.992^B^^b^	0.994^a^	0.000	**	**	**
	LL	0.994^AB^	0.994^B^	0.994^AB^	0.994					0.993^B^	0.992	0.993^A^	0.993				
	LF	0.993^B^^b^	0.993^B^^b^	0.994^B^^b^	0.995^a^					0.992^B^^c^	0.993^b^	0.993^AB^^b^	0.994^a^				
	MIX	0.993^B^	0.994^AB^	0.993^B^	0.994												

*LAB, lactic acid bacteria; SPV, sweet potato vine; CK, non-inoculated SPV silage (Control); PS, peanut straw; LL, silage inoculated with Lactococcus lactis (LL); LF, silage inoculated with Lactobacillus farciminis (LF); MIX, silage inoculated with the mixture (1:1) of LL and LF; SEM, standard error of means; D, the effect of ensiling time; T, the effect of LAB inoculation; DT, the interaction of ensiling time and LAB inoculation*.

A−B *Means in the same column followed by different uppercase letters differ (P < 0.05);*

a−c *Means in the same row followed by different lowercase letters differ (P < 0.05)*.

“* and **” denotes P < 0.05 and P < 0.01, respectively, and NS means P > 0.05.

**Figure 1:**
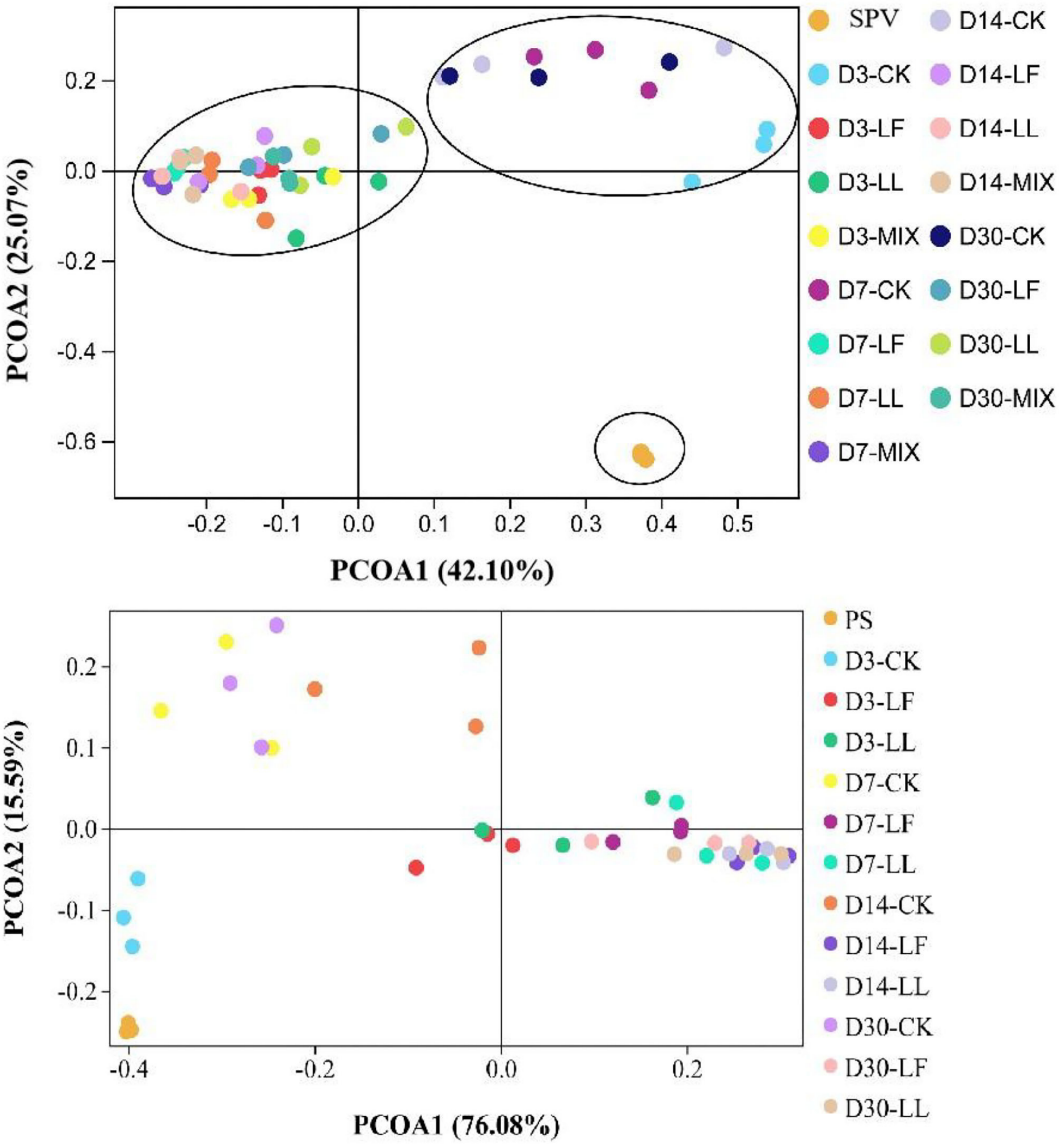
Principal coordinate analysis (PCoA) of the bacterial community of inoculated or non-inoculated SPV and PS silage. SPV, sweet potato vine; PS, peanut straw; CK, non-inoculated silage (Control); LL, silage inoculated with *Lactococcus lactis* (LL); LF, silage inoculated with *Lactobacillus farciminis* (LF); MIX, silage inoculated with the mixture (1:1) of LL and LF.

Principal coordinate analysis (PCoA, Figure 1) illustrated that the bacterial community of SPV or PS silage was differentiated apparently from that of corresponding fresh material, and inoculating LAB further led to the clear separation of bacterial community between the inoculated silage and non-inoculated silage (CK group), while the bacterial community of LL or LF inoculated silage was similar. Moreover, the bacterial community on day 3 of ensiling in the CK group was separated from those on other time-points, while the bacterial community on various time-points showed cross-distribution in inoculated silages.

**Figure 2:**
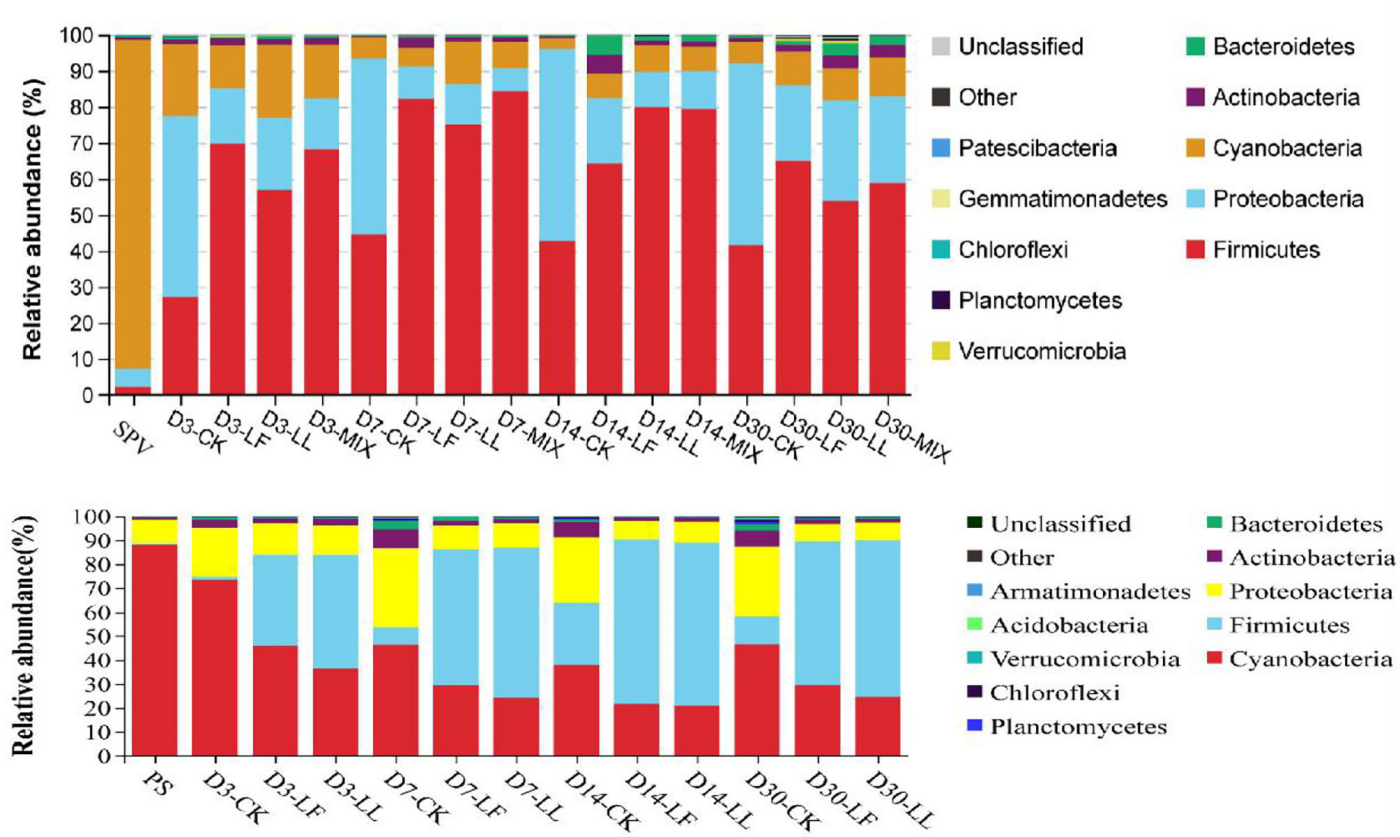
Relative abundance of the bacterial community on phylum level of inoculated or non-inoculated SPV and PS silage. SPV, sweet potato vine; PS, peanut straw; CK, non-inoculated silage (Control); LL, silage inoculated with *Lactococcus lactis* (LL); LF, silage inoculated with *Lactobacillus farciminis* (LF); MIX, silage inoculated with the mixture (1:1) of LL and LF.

As shown in Figure 2, *Cyanobacteria* was the dominant phylum (91.46, 88.67%) in the bacterial community of fresh SPV or PS, while *Firmicutes* (27.11–84.40, 0.6–68.7%), *Proteobacteria* (6.30–53.09, 7.13–33.11%), and *Cyanobacteria* (3.00–20.32, 20.71–88.02%) were the top three phyla in the silages. The relative abundance of *Proteobacteria* was lower and that of *Firmicutes* was higher in the inoculated silage relative to that of the control silage.

**Figure 3:**
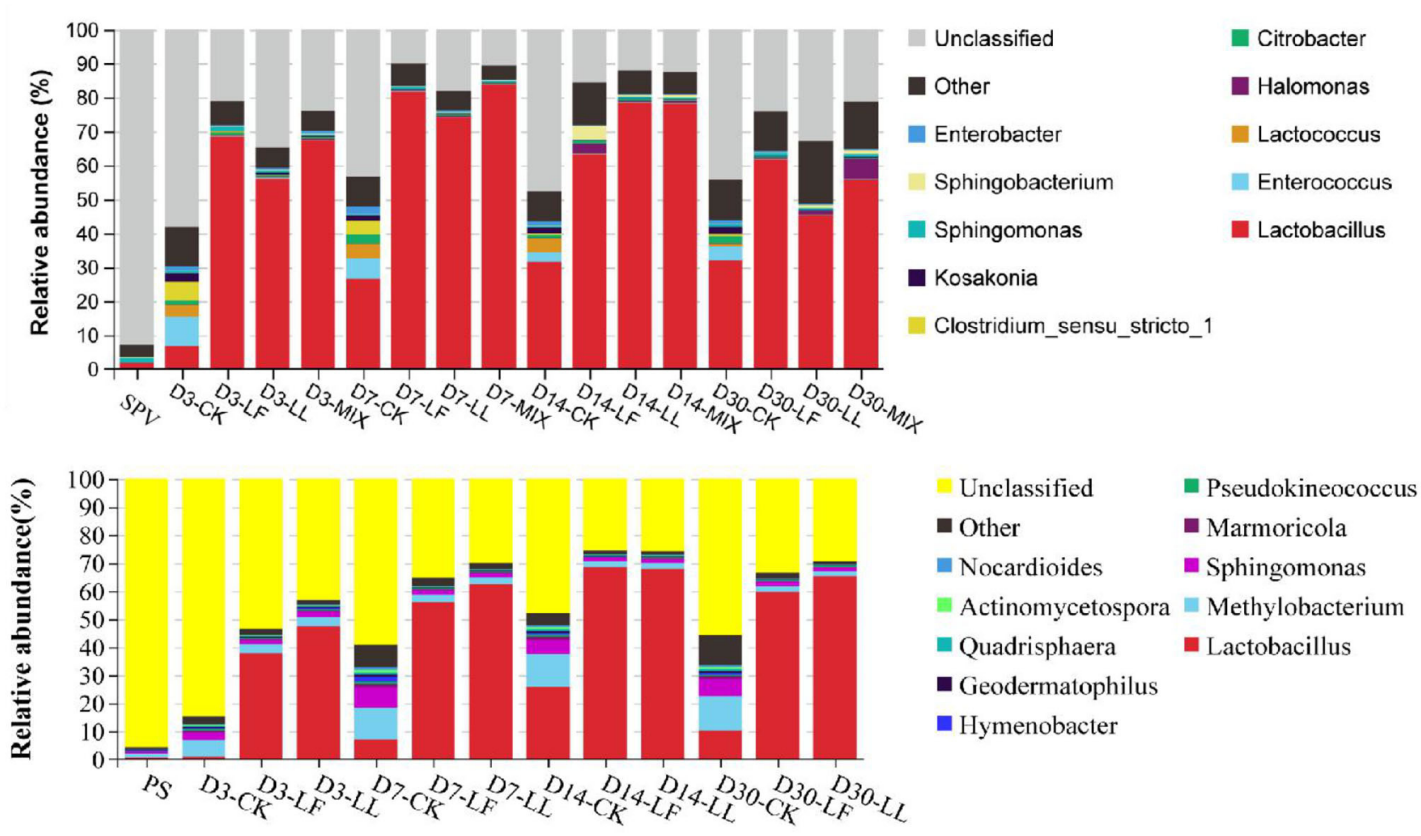
Relative abundance of the bacterial community on genus level of inoculated or non-inoculated SPV and PS silage. SPV, sweet potato vine; PS, peanut straw; CK, non-inoculated silage (Control); LL, silage inoculated with *Lactococcus lactis* (LL); LF, silage inoculated with *Lactobacillus farciminis* (LF); MIX, silage inoculated with the mixture (1:1) of LL and LF.

At the genus level (Figure 3), a remarkable difference was found in the bacterial community among the fresh material, the control silage, and the inoculated silage. In general, the majority (>90%) of the bacterial community in fresh SPV or PS was unclassified with the second generation sequencing technology of 16S rDNA, while a high proportion of the bacterial community in their silages can be identified, where the abundance of unclassified bacteria in the inoculated silage (10.66–34.68% and 27.23–52.46%) was lower than that in the control silage (43.38–58.16% and 48.04–84.23%). When focusing on the classified bacteria, the control silage was jointly dominated by several genera like *Lactobacillus, Enterococcus, Lactococcus, Clostridium, Methylobacterium*, and *Sphingomonas*. By contrast, *Lactobacillus* was the overwhelming genus in the inoculated silage since day 3 of ensiling. Meanwhile, the undesirable bacteria like *Clostridium, Enterobacter, Kosakonia, Citrobacter, Methylobacterium*, or *Sphingomonas* were much decreased in the inoculated silage.

## Discussion

### Characteristics of Raw Sweet Potato Vine and Peanut Straw

The chemical composition of SPV used in this study was in line with the results of Joo et al. (2018) reporting that SPV was a good feedstuff for ruminants containing 13.5% CP, 50.6% NDF, 33.9% ADF, and 4.68% WSC. The protein content of PS could be also high as 14% DM (Sallam et al., 2019) and PS could be used as a sole diet in goat feeding (Yusiati et al., 2016). The relatively lower protein content (9.96%) of PS in this study might be due to its lower proportion of leaf fraction or the different varieties and agronomic management.

Generally, WSC content is one of the most critical factors determining silage fermentation, and its theoretical requirement for quality silage is 60 to 70 g/kg DM (Smith, 1962). Accordingly, WSC shortage might be an issue when SPV (39.2 g/kg DM) ensiled. Moisture content is another important factor influencing silage fermentation, and a meta-analysis showed that the ideal value for high-quality silage was 65% to 70% (Guyader et al., 2018). In this study, the high-moisture content (88.72%) of SPV would make it bear the high risk of large seepage losses and *Clostridium* proliferation and decelerated pH decline, while the low moisture (51.29%) of PS might inhibit silage fermentation due to the shortage of metabolic water for the growth of LAB, and such dry silage would spoil quickly when exposed to air because of lacking sufficient amount of organic acids (e.g., acetic acid) with antifungal activity (Kung et al., 2018). Meanwhile, the high activities of coliform bacteria, yeasts, and molds might be unfavorable to the dominance establishment of LAB. In addition, bacterial community richness and diversity of fresh SPV and PS were quite different in this study. From the above, inoculating LAB might promote silage fermentation and improve nutrient preservation in SPV and PS silages.

### Ensiling Characteristics of Inoculated or Non-Inoculated SPV and PS Silages

The improper moisture content would not benefit the dominance establishment of LAB fermentation or inhibit the growth of LAB, consequently resulting in a low rate of pH decline and microbial inhibition. In this study, inoculating LAB increased initial LAB loading and promoted acid production (lactic acid), inhibiting the growth of undesirable microorganisms, thus accelerating pH decline, even though their final pH values were still slightly higher than the threshold pH 4.20 of high-quality silage (Mcdonald et al., 1991). Such an earlier steady status of silage fermentation would inhibit further DM loss in prolonged silage. It is indicated that inoculating LL or LF could improve the fermentation quality of SPV and PS silages.

In this study, the CP content of SPV silage was decreased when compared to that of fresh SPV. It might be predominantly caused by the seepage losses due to the high moisture content, which would lead to much loss of soluble N (including soluble protein and ammonia-N). Other than the gross content of CP, the fractions of CP remarkably affect its bioavailability and then determine the feeding value of feed CP. It is believed that nonprotein-N, especially ammonia-N, is less-efficient in microbial N synthesis for ruminants relative to true protein, likely increasing the nitrogen emissions in animal production (Mcdonald et al., 1991; Li et al., 2018). During the ensiling process, true protein would be inevitably degraded into nonprotein-N more or less due to the effects of plant proteases and microbial activities, thus the variation of nitrogen fractions would reflect the proteolysis extent in silage (He et al., 2019). In this study, remarkable protein hydrolysis occurred during the ensiling process, which might be interpreted as the high-moisture condition or the high-pH environment would benefit the activities of plant proteases and spoilage bacteria (e.g., proteolytic clostridia) (Muck, 2010; He et al., 2019). Inoculating LAB exerted an improvement effect on protein preservation of PS silage because inoculating LAB increased bacteria loading and lowered pH condition, which might contribute to the acid hydrolysis of protein or the activity of acid proteinase (optimal pH of 4.50 in alfalfa silage) (McKersie, 1981). The specific interpretation of these alterations needs further studies. It is inferred that the profile of raw material such as moisture content and protease activity could affect protein preservation and the effectiveness of silage additive during the ensiling process.

In principle, peptide bonds of plant protein are first hydrolyzed by plant proteases generating free amino acids and peptides (collectively termed nonprotein-N), which are further degraded into ammonia, amines, and others by the deamination of microbial activities (Kung et al., 2018; He et al., 2019). Thus, ammonia-N content could reflect the degree of protein degradation, indicating the activity of undesirable microorganisms like *Clostridium* and *Enterobacter* (Kung et al., 2018). In this study, LAB inoculation decelerated ammonia-N production and slightly decreased its proportion in mature silage of SPV silage. As aforementioned, inoculating LAB promoted acid production and inhibited the activity of spoilage microorganisms like proteolytic clostridia, thus restricting the degree of protein degradation. As ammonia-N is inferior in utilization efficiency relative to true protein and high ammonia-N content would show a negative effect on animal feed intake (Kung et al., 2018), its proportion is generally recommended as <10% TN, better lower than 5% in mature silage (Zhang et al., 2016). Furthermore, the higher nonprotein-N proportion in inoculated SPV silage did not come with a higher ammonia-N proportion, suggesting that more nonprotein-N existed in the form of amino acids or peptides. From the above, it is suggested that inoculating LAB could improve protein preservation of SPV and PS silage, with higher true protein proportion or less ammonia-N proportion.

### Bacterial Community Succession of Inoculated or Non-Inoculated SPV and PS Silage

Analyzing bacterial community succession would contribute to the interpretation of the dynamic changes of silage fermentation, which would further help to specially improve silage quality. As revealed by the alpha-diversity indices, SPV and PS silages had higher bacterial community richness and diversity relative to their raw materials, which varied during ensiling process and were altered by LAB inoculation in this study. Moreover, the alteration of the bacterial community in SPV silage was mainly reflected in community richness (Chao and Ace), while that in PS silage was community diversity (Shannon and Simpson). The Goods_coverage over 0.99 indicated that sequencing abundance was large enough to reflect the profile of the bacterial community. Inoculating LL and LF or MIX increased bacterial community richness of SPV silage but decreased community diversity of PS silage. As ensiling is a process of microbial competition, LAB inoculation would change the initial loading of LAB and its dominance establishment thereby shaping differentiated bacterial communities (He et al., 2020). In general, the greater the abundance of dominant bacteria is, the less diverse the microbial community, and vice versa. The variation in the response of SPV and PS silages might be due to the difference of epiphytic microorganisms and the chemical composition of their raw materials. Consistently, PCoA analysis showed that the bacterial community of SPV or PS silage was differentiated apparently from that of corresponding fresh material, and inoculating LAB remarkably altered the community of silage bacteria, where inoculating LAB strains LL and LF resulted in similar bacterial communities. Moreover, the bacterial community of the CK group might go through a long time to reach a steady status relative to the inoculated silage. Thus, it is believed that inoculating LAB would shape the microbial community more desirable, resulting in quality improvement. Such alterations in bacterial community might well explain the difference in fermentation quality (such as pH value and DM loss) between inoculated silage and CK silage.

The dominant phylum of the bacterial community of fresh SPV or PS was different from those of their silage. It was indicated that the bacterial community was remarkably changed on the phylum level by ensiling fermentation. Specifically, *Cyanobacteria* is the only known photosynthesizing phylum, which can use a wide nitrogen source with ammonia-N being its preferred source (Esteves-Ferreira et al., 2018), inferring that the relatively low ammonia-N content in PS silage might partly correlate with the high abundance of *Cyanobacteria*. Such bacteria might contribute to the healthy growth of the plant and have gained much attention from the pharmaceutical and biotechnical industries (Heberline, 2017). But not always the good, some *Cyanobacteria* may produce some toxins, such as microcystins, saxitoxins, nodularins, cylindrospermopsin, and anatoxin-α (He et al., 2016). Up to now, there is little study on *Cyanobacteria* in the ensiling process. Li et al. (2019) reported that *Cyanobacteria* was the main phylum in pre-ensiled king grass, paspalum, white popinac, and stylo, but not in the mature silage. The role of *Cyanobacteria* in the ensiling process still needs further study.

Consistently, Ogunade et al. (2018) concluded in a review that the majority of the bacterial community in silage belonged to the phylum *Firmicutes* and *Proteobacteria*. In this study, inoculating LAB dramatically altered the bacterial communities of SPV and PS silages. The decline of *Proteobacteria* might be attributed to the low pH condition, in that *Proteobacteria* were reported to prefer the neutral environment (Brenner et al., 2005). *Proteobacteria* might play a crucial role in nitrogen cycling given that Li et al. (2020) reported that the abundance of *Proteobacteria* was positively correlated with ammonia-N content in wastewater fermentation. It is inferred that the improvement of protein preservation might somewhat correlate with the alteration of *Proteobacteria*.

The majority (>90%) of the bacterial community in fresh SPV or PS could not be classified on genus level with the second-generation sequencing technology of 16S rDNA, while a high proportion of the bacterial community in their silages can be identified based on the current database. It might be due to the relatively poor development of *Cyanobacterial* taxonomy, where most of the *Cyanobacteria* cannot be cultured in the present knowledge (Palinska and Surosz, 2014). Even though differentiated bacterial communities were illustrated, a higher annotated level of sequencing technology such as PacBio full-length 16S rDNA sequencing might further improve the analysis.

When focusing on the classified bacteria, the control silage was jointly dominated by several genera, showing that LAB could not dominate the silage till the end. By contrast, *Lactobacillus* was the overwhelming genus in the inoculated silage since day 3 of ensiling. It was confirmed that inoculating LAB did promote the dominance establishment of LAB during ensiling fermentation, consequently resulting in more acid production and faster pH decline. *Lactococcus, Enterococcus, Leuconostoc, Weissella*, and *Lactobacillus* are common lactate-producing bacteria in silage (Pahlow et al., 2003), where less acid-tolerant cocci like *Lactococcus* and *Enterococcus* initiate lactic acid fermentation at the early stage of ensiling and the more acid-tolerant bacilli like *Lactobacillus* dominate the community later (Cai et al., 1998). Meanwhile, the undesirable bacteria were much decreased in the inoculated silage. *Enterobacter* and *Clostridium* are undesirable bacteria in silage fermentation in that their activities would cause much protein degradation, dry matter loss, ammonia and butyric acid production, discounting acid fermentation, and pH decline (Pahlow et al., 2003; Muck, 2010). *Kosakonia* is a new genus recently classified from the genus *Enterobacter* (Li, 2016). *Methylobacterium* is strictly aerobic, neutrophilic, and facultative methylotrophic bacteria commonly found in plants (Doronina et al., 2002), and their abundance is reported to positively correlate with silage pH (Ogunade et al., 2018). Its relative high abundance in PS silage should be ascribed to the air residue and high-pH condition caused by the low-moisture content. *Sphingomonas*, belonged to Gram-negative aerobic *Alpha-proteobacteria*, are also detected in agricultural byproducts silage and are considered to cause hydrolysis of soluble protein (Zhou et al., 2019). So their increased abundance in the silage might be undesired and their exact roles in silage fermentation need further research. From the above, inoculating LAB remarkably enlarged the relative abundance of LAB, decreased the abundance of undesirable bacteria, and accelerated the dominance establishment of LAB in the bacterial community of SPV and PS silages.

## Conclusions

The results showed that inoculating screened LAB strains LL and LF increased lactic acid production and accelerated pH decline, and decreased butyric acid and nonprotein-N or ammonia-N content in SPV and PS silages. Meanwhile, it remarkably altered the bacterial community of the silages, where the relative abundance of beneficial bacteria *Lactobacillus* was largely increased and that of undesirable bacteria such as *Clostridium, Enterobacter, Methylobacterium*, or *Sphingomonas* was much decreased. It is suggested that inoculating screened LAB strains LL and LF can dramatically improve the silage quality of SPV and PS silages.

## Data Availability Statement

The datasets presented in this study can be found in online repositories. The name of the repository and accession number can be found below: NCBI; PRJNA812632 and PRJNA813727.

## Author Contributions

LH: conceptualization, methodology, and writing—reviewing and editing. YW: investigation and data curation and manuscript checking. XG: methodology and investigation. XC: validation and supervision. QZ: conceptualization, methodology, and visualization. All authors contributed to the article and approved the submitted version.

## Funding

This work was financially supported by the China Agriculture Research System of MOF and MARA (CARS-39), Guangzhou Science and Technology Bureau Project (202102020808), and Guangdong Natural Science Foundation (2020A1515011253).

## Conflict of Interest

The authors declare that the research was conducted in the absence of any commercial or financial relationships that could be construed as a potential conflict of interest.

## Publisher's Note

All claims expressed in this article are solely those of the authors and do not necessarily represent those of their affiliated organizations, or those of the publisher, the editors and the reviewers. Any product that may be evaluated in this article, or claim that may be made by its manufacturer, is not guaranteed or endorsed by the publisher.
